# Narrow UVB-Emitted YBO_3_ Phosphor Activated by Bi^3+^ and Gd^3+^ Co-Doping

**DOI:** 10.3390/nano13061013

**Published:** 2023-03-11

**Authors:** Zhimin Yu, Yang Yang, Jiaming Sun

**Affiliations:** School of Materials Science and Engineering, Tianjin Key Lab for Rare Earth Materials and Applications, Nankai University, Tianjin 300350, China

**Keywords:** UVB emission, rare-earth orthoborate, gadolinium, phosphor, co-doping

## Abstract

Y_0.9_(Gd_x_Bi_1−x_)_0.1_BO_3_ phosphors (x = 0, 0.2, 0.4, 0.6, 0.8, and 1.0, YGB) were obtained via high-temperature solid-state synthesis. Differentiated phases and micro-morphologies were determined by adjusting the synthesis temperature and the activator content of Gd^3+^ ions, verifying the hexagonal phase with an average size of ~200 nm. Strong photon emissions were revealed under both ultraviolet and visible radiation, and the effectiveness of energy transfer from Bi^3+^ to Gd^3+^ ions was confirmed to improve the narrow-band ultraviolet-B (UVB) (^6^P_J_→^8^S_7/2_) emission of Gd^3+^ ions. The optimal emission was obtained from Y_0.9_Gd_0.08_Bi_0.02_BO_3_ phosphor annealed at 800 °C, for which maximum quantum yields (QYs) can reach 24.75% and 1.33% under 273 nm and 532 nm excitations, respectively. The optimal QY from the Gd^3+^-Bi^3+^ co-doped YGB phosphor is 75 times the single Gd^3+^-doped one, illustrating that these UVB luminescent phosphors based on co-doped YBO_3_ orthoborates possess bright UVB emissions and good excitability under the excitation of different wavelengths. Efficient photon conversion and intense UVB emissions indicate that the multifunctional Gd^3+^-Bi^3+^ co-doped YBO_3_ orthoborate is a potential candidate for skin treatment.

## 1. Introduction

Skin treatment using artificial sources of ultraviolet (UV) radiation in controlled conditions is well established, and narrow-band ultraviolet-B (UVB) therapy has been demonstrated to be effective against skin diseases and disorders such as psoriasis, vitiligo and hyperbilirubinemia (commonly known as infant jaundice) [1,2,3,4]. Phototherapy with narrow-band UVB (310–313 nm) as photosensitizers is believed to result from the direct interaction between the light of certain frequencies and tissues, causing a change in immune response [5,6,7]. Furthermore, during phototherapy investigations, it was observed that light belonging to longer wavelengths of the UVB region was more effective, while that of the shorter wavelengths was much less effective or even harmful [8,9]. Rare-earth (RE) orthoborates (RE-BO_3_, RE = lanthanide, yttrium, and scandium) have aroused considerable interest due to their wide range of applications in plasma display panels and mercury-free fluorescent lamps [10,11]. In particular, YBO_3_ is an excellent host for UV phosphors due to its high-vacuum UV transparency, exceptional optical damage thresholds, strong absorption in the UV range, and good chemical inertness [12,13,14]. Additionally, the YBO_3_ phosphors exhibit a wide bandgap and high host-to-activator energy transfer efficiency at moderate RE^3+^ concentrations [15]. Therefore, it is of great interest to investigate RE-doped YBO_3_ orthoborates for UVB treatments.

**Figure 1:**
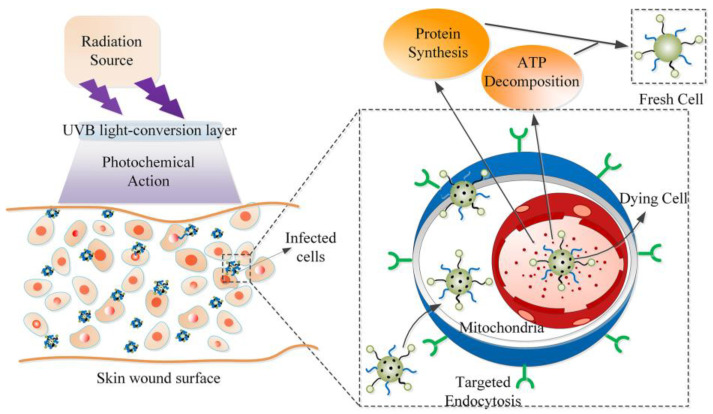
Scheme of light conversion layer for skin treatment using UVB emissions.

Among the RE ions, lanthanide gadolinium (Gd^3+^) is of particular interest because of its ubiquitous nature (well known as U-spectrum) and the characteristic narrow-band UVB emission from ^6^P_J_→^8^S_7/2_ transitions [16,17]. The optical properties of Gd^3+^ ions have been widely studied, and many Gd-doped compounds can be used as efficient phosphors in the new generation of UV fluorescent lamps. Moreover, as a promising activator or sensitizer, the Bi^3+^ ion shows excellent emission and absorption ability in the UV region. Furthermore, the transitions of ^8^S_7/2_→^6^I_J_ (emission at ~270 nm) and ^8^S_7/2_→^6^P_J_ (at ~311 nm) of the Gd^3+^ ion overlap with the ^3^P_1_→^1^S_0_ (at ~260 nm) transition of the Bi^3+^ ion in YBO_3_ [18,19], which permits an efficient energy transfer from the Bi^3+^ to Gd^3+^ ions. Further research on improving the UVB emission has also been reported [18]. From a practical point of view, UV-emitting phosphors in well-defined regions are required for various applications. Keeping this in mind, we prepared a UVB-emitting Gd^3+^-Bi^3+^ co-doped YBO_3_ phosphor in this work, which can effectively achieve light conversion and UVB emissions. When UV fluorescence is irradiated on the surface of a skin wound, the activity of the mitochondrial catalase can increase in cells, which could promote the synthesis of proteins and the decomposition of adenosine triphosphate (ATP), ultimately healing the wound. A schematic representation of healing, which adopts phosphors as light conversion layers, is conceived in Figure 1.

In this work, narrow-band UVB-emitting phosphors of Gd^3+^-Bi^3+^ co-doped Y_0.9_(Gd_x_Bi_1−x_)_0.1_BO_3_ (YGB, x = 0, 0.2, 0.4, 0.6, 0.8 and 1.0) were fabricated by high-temperature solid-state synthesis. The samples with the hexagonal phase and well-dispersed particles were characterized by XRD and SEM techniques, manifesting a microsize of ~200 nm. The responses to UV and the visible (VIS) radiation of these YGB phosphors were compared, and sharp UVB luminescence was recorded with the adjustment of Gd^3+^ content. The sintering temperature indicated that co-doped Bi^3+^ ions enhanced the characteristic UVB luminescence from Gd^3+^ ions. The spectroscopic intensity parameters of YGB phosphors were derived from relative spectral power distributions, and the maximum quantum yields (QYs) at 313 nm were calculated at 24.75% and 1.33% under 273 nm and 532 nm excitations, respectively. YGB orthoborate phosphors with intense UVB emission could provide a viable approach for developing multifunctional composite materials for skin treatments.

## 2. Materials and Methods

The powders of Y_0.9_(Gd_x_Bi_1−x_)_0.1_BO_3_ (x = 0, 0.2, 0.4, 0.6, 0.8, and 1.0, marked as YGB-0, YGB-0.2, YGB-0.4, YGB-0.6 YGB-0.8, and YGB-1.0, respectively) phosphors were prepared using high-purity reagents Y_2_O_3_ (99.9%), Gd_2_O_3_ (99.9%), Bi_2_O_3_ (A.R.), and H_3_BO_3_ (A.R.) as raw materials. The original chemicals for YGB with different Bi^3+^ contents as the designed sensitizer were mixed by grinding them in an agate mortar according to the stoichiometric ratio. The raw powders were transferred into alumina crucibles and pre-sintered at 500 °C for 1 h and then sintered at 700 °C, 800 °C, 900 °C and 1000 °C for 5 h. Afterwards, the samples were ground thoroughly after cooling.

**Figure 2:**
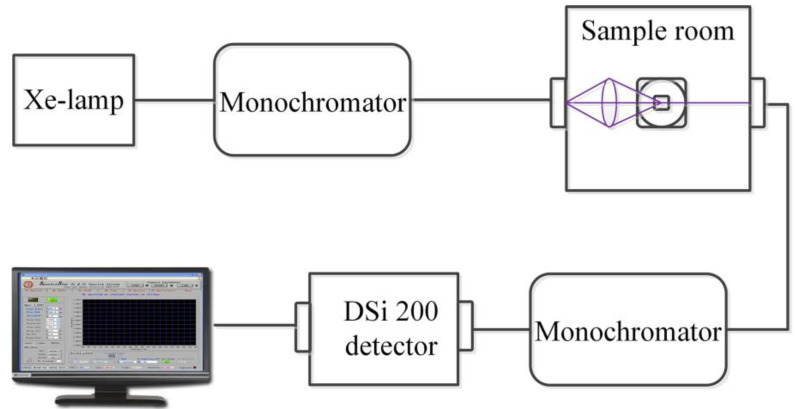
The schematic diagram of the experimental setup for the PL measurement.

The phase and the crystal structure of powders were identified by an X-ray diffractometer (XRD, MiniFlex 600, Rigaku, Tokyo, Japan) using Cu Kα radiation. Morphologies of the powders were analyzed by a field emission scanning electron microscope (SEM, JSM-7800F, JEOL, Tokyo, Japan) equipped with energy dispersive spectroscopy (EDS, X-MaxN 50, Oxford, Oxford, UK) using an accelerating voltage of 15 kV. Particle size distributions were measured in a Nanoparticle Analyzer (Zetasizer Nano-ZS, Malvern, UK). Photoluminescence (PL) spectra were recorded using a Keithley 2010 multimeter and the monochromator (λ500, Zolix, Beijing, China) equipped with a Si detector (DSi200, Zolix, Beijing, China). A commercial Xe lamp and two solid-state lasers emitting at 266 nm and 532 nm were used as the excitation sources for different excitation wavelengths. A standard PTFE diffuse reflective white plate (reflectivity greater than 99.9%) was used as a reference. The schematic diagram of the experimental setup is depicted in Figure 2. The relative spectral power distribution was obtained and calibrated by the Optical Power Meter (1830-C, Newport, Newport County, RI, USA). All measurements were performed at room temperature.

## 3. Results

### 3.1. Structure and Morphology

**Figure 3:**
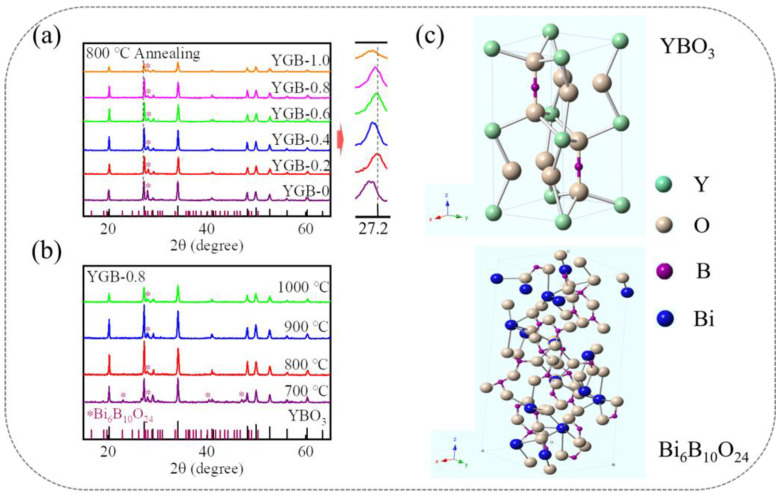
XRD patterns of (**a**) Y_0.9_(Gd_x_Bi_1−x_)_0.1_BO_3_ (x = 0, 0.2, 0.4, 0.6, 0.8 and 1.0) phosphors annealed at 800 °C and (**b**) YGB-0.8 phosphors with different annealing temperatures ranging from 700 °C to 1000 °C. The ⁎ represents the diffraction peaks from Bi_6_B_10_O_24_ phase. (**c**) The crystal structure of YGB systems, showing the coordination environment of YBO_3_ and Bi_6_B_10_O_24_.

Figure 3a shows the XRD patterns of YGB phosphors with different compositions annealed at 800 °C, the main peaks of which are in agreement with the JCPDS Card of YBO_3_ (PDF#16-0277). The phosphors were confirmed as polycrystalline materials that possess a hexagonal crystal structure with space group P63/m and the cell parameters of a = b = 3.778 Å and c = 8.81 Å, similarly to what has been previously reported [20]. Moreover, the well-defined sharp diffraction peaks imply that these samples have high crystallinity, illustrating that Gd^3+^ and Bi^3+^ ions are substituted within the host. Some small impurity peaks are identified as Bi_6_B_10_O_24_ (PDF#29-0228), which are attributed to the interaction of Bi_2_O_3_ and excess H_3_BO_3_ during the fabrication process [21]. The corresponding reaction equation is as follows: 2Bi_2_O_3_ + B_2_O_3_→Bi_4_B_2_O_9_ and 3Bi_4_B_2_O_9_ + 7B_2_O_3_→2Bi_6_B_10_O_24_ [22]. Here, the XRD peaks (2θ = ~27.2°) of YGB samples with slightly smaller angles, in comparison with the standard YBO_3,_ should be attributed to the larger radius of Gd^3+^ (1.053 Å, 8-coordination) and Bi^3+^ (1.170 Å, 8-coordination) relative to that of Y^3+^ (1.019 Å, 8-coordination), according to Bragg’s law [23,24,25], while the shift in diffraction peaks resulted from the different Gd^3+^-Bi^3+^ contents in these phosphors. The slight change is also attributed to the surface charge redistribution of the crystal nucleus, induced by an inner-electron charge transfer between the doped ions and lattice cations [26,27]. Therefore, these results show that doped Gd^3+^ and Bi^3+^ ions do not affect the main crystal structure of YBO_3_ and should be completely dissolved into the host lattice.

As shown in Figure 3b, the impurities decrease, and the relative intensities of diffraction peaks initially increase and then decrease with the annealing temperature; this is attributed to the melting point of Bi_6_B_10_O_24_ and the selectivity of the growth in the solid-state synthesis process [25,28]. In addition, the YGB crystal is composed of 8-coordinated Y^3+^ and 4- coordinated B^3+^ ions, which is illustrated in Figure 3c. Here, the Y^3+^ ions are 8- coordinated with two nonequivalent environments, while the B^3+^ ions and two interconnected BO_4_ tetrahedral coordination form (BO_3_)^3−^ groups [29]. Moreover, due to the similar ionic radii of the 8-coordinated Y^3+^, Bi^3+^, and Gd^3+^ ions, Gd^3+^ and Bi^3+^ ions can easily substitute the Y^3+^ sites and form a solid solution of (Y,Gd,Bi)BO_3_ crystals.

**Figure 4:**
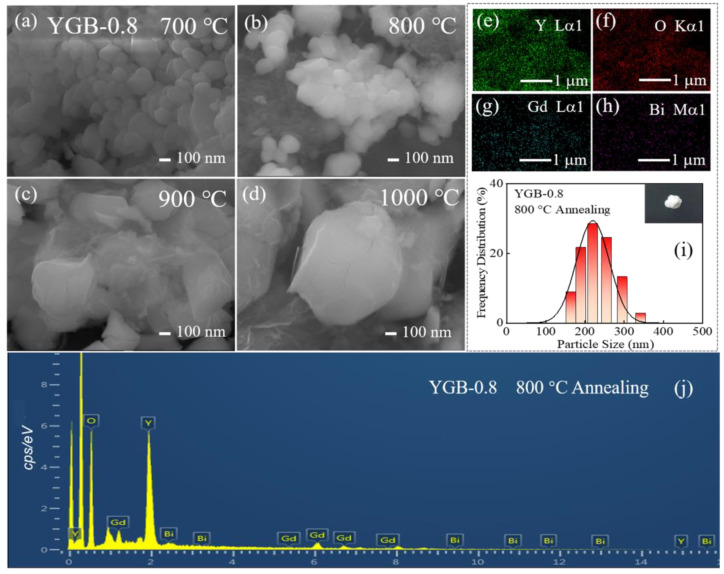
(**a**–**d**) SEM images of YGB-0.8 samples with different annealing temperatures ranging from 700 °C to 1000 °C. (**e**–**h**) Elemental mapping; (**i**) particle size distributions and (**j**) EDS spectrum of the YGB-0.8 sample annealed at 800 °C. Inset in (**i**): the macroscopic appearance of the sample exhibited under natural light irradiation.

Take the Y_0.9_Gd_0.08_Bi_0.02_BO_3_ (YGB-0.8) phosphor as an example. The SEM images in Figure 4a–d show the typical morphologies of particles annealed at 700, 800, 900, and 1000 °C, revealing that the powders annealed at 800 °C and below possess an average size of ~200 nm and the regular morphology. To show this, the particle size distributions of the YGB-0.8 phosphor annealed at 800 °C are shown in Figure 4i. Here, the inset shows the macroscopic appearance of the sample exhibited under natural light irradiation. It can be observed that the particle size is mainly concentrated at ∼200 nm, which is consistent with the SEM images. As shown in the SEM images, the powders with a narrow particle size distribution have been synthesized at lower sintering temperatures, and they possess a large effective surface area and weak atomic binding energy, resulting in the lower local symmetry of the YO_8_ polyhedron and the surface defects of nanoparticles. When the annealing temperature exceeds a certain value, the crystal phase is gradually purified together with grain growth. Obviously, the morphology becomes more irregular in angularity, heterogeneity and compactness with the increase in sintering temperature, which is due to the changes in van der Waals attractions, while the small particle size may be caused by the distortion of anionic groups on the particle’s surface [30,31,32]. The size of spherical particles is significantly larger after sintering at 1000 °C, while compositional particles lose their spherical shape and undergo significant aggregation, which is attributed to the higher activity of atoms on the particle’s surface caused by the further decomposition of precursors. Under higher temperature annealing, the atoms could diffuse and combine with adjacent ones to form stable chemical bonds, leading to agglomeration [33,34]. No obvious changes in the morphology or particle size with various Gd^3+^ contents were observed at the same sintering temperature (not shown here), indicating that the doped Bi^3+^ and Gd^3+^ ions do not impact crystallization and grain growth. For the YGB-0.8 sample annealed at 800 °C, the homogeneous distributions of Gd, Bi, O, and Y elements are clearly observed by EDS, as shown in Figure 4e–h,j. The B element is undetected since its corresponding energy in the X-ray spectrum falls outside the scope. Moreover, high-packing densities, good slurry properties, and well-distributed particles in YGB systems are conducive to photon release.

### 3.2. Fluorescence Behaviors of YGB Phosphor

**Figure 5:**
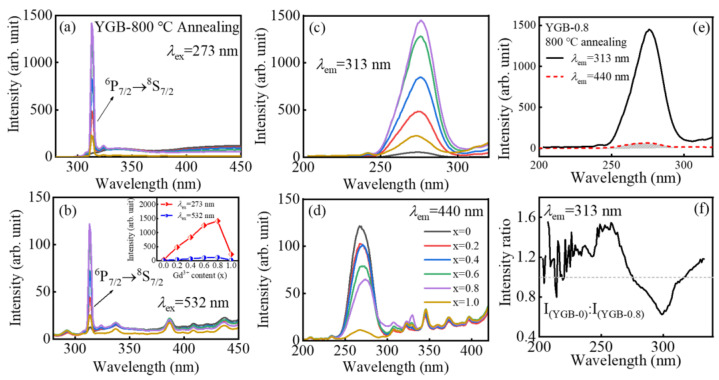
PL spectra of YGB phosphors (x = 0, 0.2, 0.4, 0.6, 0.8, and 1.0) under the (**a**) 273 nm and (**b**) 532 nm excitations, and their PLE spectra monitoring at (**c**) 313 nm and (**d**) 440 nm. Inset in (**b**): The dependence of PL emission intensities at 313 nm on the Gd^3+^ content (x) in YGB phosphors under 273 nm and 532 nm excitations. (**e**) Excitation spectra of YGB-0.8 phosphor with the spectral overlap presented by the shade and (**f**) the fluorescence intensity ratio of the 800 °C annealed YGB-0.8 and YGB-0 phosphors monitored at 313 nm.

Figure 5a,b show the typical emission spectra of YGB phosphors with different Bi^3+^/Gd^3+^ contents (x = 0, 0.2, 0.4, 0.6, 0.8 and 1.0) under UV (273 nm) and VIS (532 nm) excitations. Notably, strong UV emissions can also be obtained by up-conversion under the excitation of a 532 nm laser, and all samples show a narrow-band emission at 313 nm, which is attributed to the ^6^P_7/2_→^8^S_7/2_ transition of Gd^3+^ ions [6]. Emission intensity increases until the Gd^3+^ content exceeds x = 0.8, which results from the energy transfer between Bi^3+^ and Gd^3+^ ions; with the further increase in Gd^3+^ contents, concentration quenching occurs with the attenuation of emission intensity. In order to identify the change in spectral intensity more clearly, the dependence of the PL emission intensity at 313 nm on the Gd^3+^ content (x) in YGB phosphors under 273 nm and 532 nm excitations is illustrated in the inset of Figure 5b. Compared with the sample without Bi^3+^, the weaker wide emission located around 440 nm resulted from the 6s^2^→6s6p transitions of Bi^3+^ ions. According to the photoluminescence excitation (PLE) spectra of these YGB phosphors in Figure 5c, monitored at 313 nm, the strongest excitation band centered at 273 nm should be attributed to the ^8^S_7/2_→^6^I_J_ transition of the Gd^3+^ ion, which well overlaps with the 253.7 nm line of mercury lamps [35], while the emission peak at 440 nm was derived from the ^3^P_1_→^1^S_0_ transition of Bi^3+^ ions [36]. In particular, Figure 5d shows the PLE spectra of YGB samples with different Gd^3+^ contents in monitoring the 440 nm emission, the intensity of which decreases with the Gd^3+^ content, illustrating the energy transfer (ET) from Bi^3+^ to Gd^3+^ ions that consumes the excitation energy of Bi^3+^ ions.

The energy transfer depends on the overlap between the excitation band of the activator and the emission band of the sensitizer in the phosphors. Bi^3+^ ions have a 6s^2^ outer electronic configuration with a ^1^S_0_ ground state, and the excited state has the configuration of 6s6p with ^3^P_0_, ^3^P_1_, ^3^P_2_, and ^1^P_1_ splitting levels. Due to the forbidden transitions of ^1^S_0_→^3^P_0_ and ^1^S_0_→^3^P_2_ by the electronic selection rules, the ^1^S_0_→^3^P_1_ and ^1^S_0_→^1^P_1_ transitions of Bi^3+^ ions are usually observed [37]. For the sample doped with only Bi^3+^ ions, it would first relax and transit into the lowest ^3^P_1_ excited state and then return to the ^1^S_0_ ground state via radiation. However, when Bi^3+^ and Gd^3+^ ions were co-doped into the host, energy transfer would occur, since the ^3^P_1_→^1^S_0_ emission of Bi^3+^ effectively overlapped with the energy levels of Gd^3+^ (^6^P_7/2_, ^6^P_5/2_, and ^6^P_3/2_) [38]. For the Y_0.9_Gd_0.08_Bi_0.02_BO_3_ (YGB-0.8) sample annealed at 800 °C, the overlapped excitation spectra of Bi^3+^ and Gd^3+^ ions are shown in Figure 5e, confirming their efficient excitability in the short-wave UV region, which is advantageous for the resonance energy transfer from Bi^3+^ to Gd^3+^ ions. In order to further clarify the controversies over the ET from Bi^3+^ to Gd^3+^, Figure 5f presents the Gd^3+^ fluorescence intensity ratio between 800 °C annealed YGB-0 and YGB-0.8 phosphors, monitored at 313 nm, which demonstrates the sensitizing effect of Bi^3+^ on Gd^3+^ ions. Compared with the sample without Gd^3+^ ions, the excitation energy of Bi^3+^ in Bi^3+^-Gd^3+^ co-doped samples is transferred to the Gd^3+^ ion and leads to stronger fluorescence emissions from the Gd^3+^ ion in short-wave UVB radiation, resulting in increased excitability. In addition, the excitation peaks located at 258 nm are consistent with the characteristic excitation peaks of Bi^3+^ (^1^S_0_→^1^P_1_) [25,37], which further confirms the effectiveness of ET from Bi^3+^ to Gd^3+^ ions.

**Figure 6:**
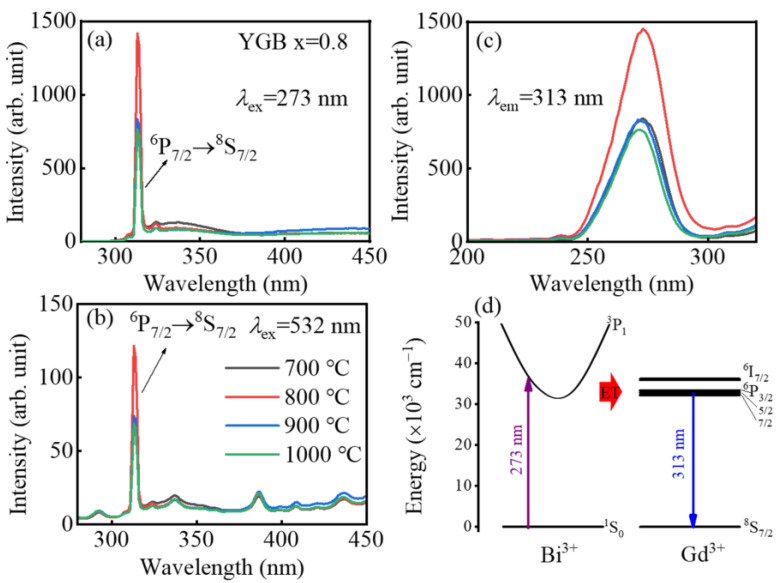
PL spectra of YGB-0.8 phosphors annealed from 700 °C to 1000 °C under (**a**) 273 nm and (**b**) 532 nm excitations. (**c**) Their PLE spectra monitored at 313 nm and (**d**) the schematic energy transfer mechanism from Bi^3+^ to Gd^3+^ ions.

To further investigate the effect of sintering temperatures on luminous properties, the PL and PLE spectra measured at room temperature from the YGB-0.8 phosphors annealed at different temperatures are illustrated in Figure 6a–c. The intensity of the excitation peak at 313 nm increases with the annealing temperature until 800 °C. The grain size increases while the porosity decreases significantly with the increase in temperature, enhancing the luminous intensity. When the sintering temperature is higher than 800 °C, the decreased intensity is attributed to the accelerated volatilization of Bi^3+^ and the crystalline defects. Furthermore, the enhanced PL emission originates from the absorption of exciting UV light by co-doped Bi^3+^ ions, which transfer the energy to the Gd^3+^ ions [18,39]. This mechanism is schematically shown in Figure 6d. Firstly, phosphors absorb the UV light, which leads to the ^1^S_0_→^3^P_1_ transition of Bi^3+^ ions. The Bi^3+^ ions then transfer the energy non-radiatively to Gd^3+^ ions and ultimately realize UVB emissions from Gd^3+^ ions. Moreover, the smaller electronegativity of Gd^3+^ (1.20), compared to that of Y^3+^ (1.22) and Bi^3+^ ion (~2.02), allows an easier charge transfer, thus promoting PL emissions [19,40].

**Figure 7:**
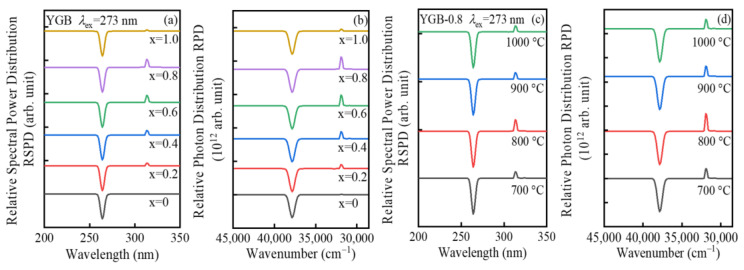
(**a**,**c**) Relative spectral power distributions and (**b**,**d**) relative photon distributions of Y_0.9_(Gd_x_Bi_1−x_)_0.1_BO_3_ (x = 0, 0.2, 0.4, 0.6, 0.8 and 1.0) and YGB-0.8 samples with different annealing temperatures from 700 °C to 1000 °C under the 273 nm excitation.

In order to evaluate the optical property of Gd^3+^-Bi^3+^ co-doped YGB phosphors with different Gd^3+^ contents and annealing temperatures, the relative spectral power distributions and relative photon distributions were determined and compared, as in Figure 7a–d. Under the 273 nm excitation, the sharp narrow-band UVB emission at 313 nm that originates from the ^6^P_J_→^8^S_7/2_ transition increases significantly with the increase in Gd^3+^ content and annealing temperature, which reaches the maximum value while the Gd^3+^ content is x = 0.8 and is annealed at 800 °C. This should be attributed to the increased energy transfer caused by the reduced distance among Gd^3+^-Gd^3+^ ion pairs [41]. The relative photon distribution provides fundamental information with respect to optical fields and relevant applications. Depending on the relative spectral power distribution ***P***(*λ*), photon distribution ***N***(*ν*) can be deduced by $N\left(\nu \right)=\frac{\lambda^{3}}{hc}P(\lambda )$, where *ν*, *λ*, *h*, *c*, and ***P***(*λ*) represent wavenumber, wavelength, Planck constant, vacuum light velocity, and spectral power distribution, respectively [42]. Here, the abscissae of the distribution spectra were converted to a wavenumber (cm^−1^) for accurate deconvolution. The net absorption and emission photon distribution curves of Gd^3+^-Bi^3+^ co-doped YBO_3_ phosphors were derived, as presented in Figure 7b,d, and their net emission and absorption intervals were selected at 30,300–33,300 cm^−1^ (corresponding to 313 nm) and 36,300–39,200 cm^−1^ (corresponding to 273 nm).

**Figure 8:**
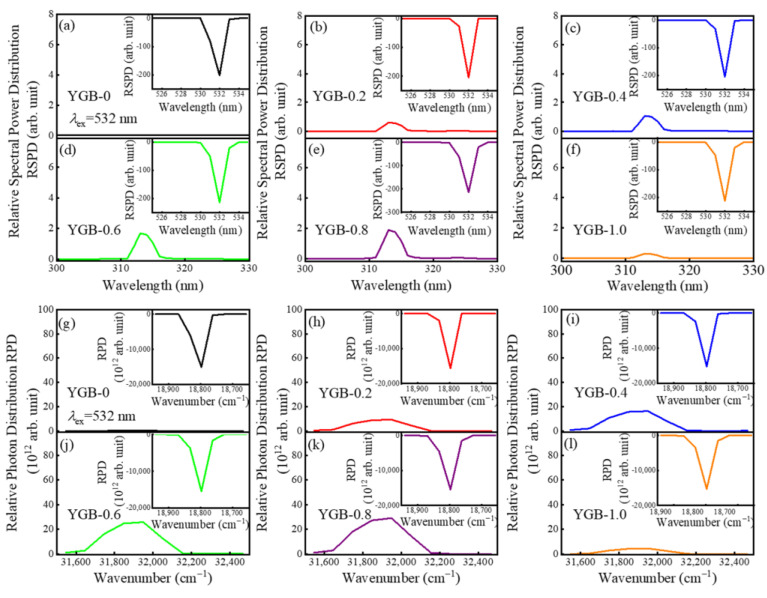
(**a**–**f**) Relative spectral power distributions and (**g**–**l**) relative photon distributions of Y_0.9_(Gd_x_Bi_1−x_)_0.1_BO_3_ (x = 0, 0.2, 0.4, 0.6, 0.8 and 1.0) phosphors annealed at 800 °C under the 532 nm excitation.

In addition, upon the excitation under 532 nm VIS light, the samples still emit the up-conversion UVB emission at 313 nm. The spectral power distribution and the photon number distribution of all samples with different Gd^3+^ contents (x = 0, 0.2, 0.4, 0.6, 0.8, and 1.0) are displayed in Figure 8. With the increase in Gd^3+^ contents, the emission intensity increases until Y_0.9_Gd_0.08_Bi_0.02_BO_3_, because the transfer probability is proportional to the interaction between the sensitizer (Bi^3+^) and the activator (Gd^3+^) in both non-radiative and radiative resonance energy transfers. When x > 0.8, the intensity significantly decreases due to prominent concentration quenching caused by the reduced distance among Gd^3+^-Gd^3+^ ions.

**Figure 9:**
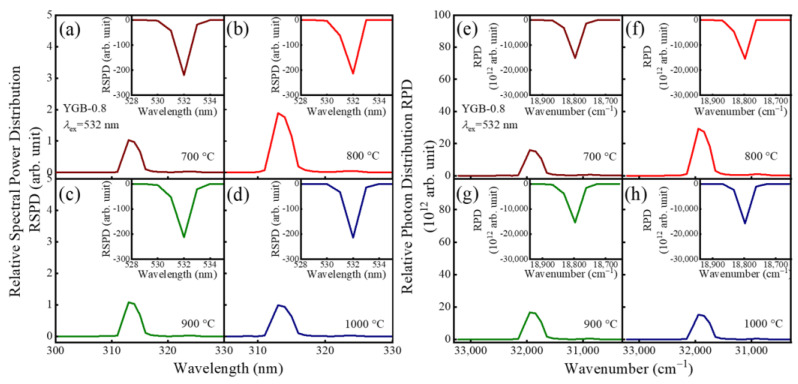
(**a**–**f**) Relative spectral power distributions and (**e**–**h**) relative photon distributions of YGB-0.8 samples with different annealing temperatures from 700 °C to 1000 °C under the 532 nm excitation.

According to Blasse [43,44], the energy would transfer from one activator to another until all energy is consumed. This phenomenon is regarded as concentration quenching in fluorescence, which is due to the non-radiative energy transfer among identical ions. Thus, the critical distance (*R_C_*) is a parameter that is essential to understanding this phenomenon, which is calculated using the following equation: $R_{c}=2{\left[\frac{3V}{4\pi x_{c}N}\right]}^{\frac{1}{3}}$, where *V* is the volume of the unit cell (in Å^3^), *x_c_* is critical concentration, and *N* is the number of Y^3+^/Bi^3+^/Gd^3+^ ions in the unit cell. Herein, the values are *x_c_* = 0.08, *N*= 6, and *V* = 108.90 Å^3^, and the critical distance *R_C_* of the YGB phosphor is calculated to be about 7.57 Å. Meanwhile, the corresponding spectral power distribution and photon number distribution of YGB-0.8 phosphors annealed from 700 °C to 1000 °C under the 532 nm excitation were also derived, and they are shown in Figure 9 to demonstrate the up-conversion emission monitored at 313 nm and the optimal annealing temperature of 800 °C. These results verify the effectiveness of Gd^3+^-Bi^3+^ co-doped phosphors in photon conversion and provide the theoretical basis for their application in skin treatments.

The spectral parameters could also provide external quantum yields (QYs) to assess luminescence and laser materials, which are used to calculate the utilization efficiency of the absorbed photons for desired emissions, defined as the photon number ratio of emission and absorption. Namely, QY = emitted photons/absorbed photons = N_em_/N_abs_. Here, the maximum QY is derived to be 24.75% in a Y_0.9_Gd_0.08_Bi_0.02_BO_3_ sample annealed at 800 °C under the 273 nm excitation, which is larger than that of other Gd^3+^ ions doped phosphors [45,46], and it is 75 times the single Gd^3+^-doped sample in this work. On the basis of these QYs, a higher photon release efficiency is achieved, which further exhibits the potential of Gd^3+^-Bi^3+^ co-doped YBO_3_ phosphors for UVB skin treatment and reflects the energy transfer effectiveness between Bi^3+^ and Gd^3+^ ions in these phosphors. Moreover, this phosphor maintains a unique up-conversion excitability in the VIS region with a QY of 1.33% under the excitation of 532 nm. The QY values for the different contents and annealing temperatures of these co-doped YGB phosphors, under the excitation of the 273 and 532 nm, are listed in Table 1. These results reveal that the Gd^3+^-Bi^3+^ activated YBO_3_ phosphors with up/down-conversion excitability exhibit excellent UVB emission performance.

## 4. Conclusions

UVB-emitting Y_0.9_(Gd_x_Bi_1−x_)_0.1_BO_3_ phosphors (x = 0, 0.2, 0.4, 0.6, 0.8, and 1.0) with the hexagonal phase and an average ~200 nm grain size were fabricated via the solid-state synthesis method. The enhanced PL emissions and the overlapped spectra verify the energy transfer between the Bi^3+^ and Gd^3+^ ions and a well-defined sharp and intense peak centered at 313 nm due to the ^6^P_7/2_→^8^S_7/2_ transitions of Gd^3+^ ions. The Y_0.9_Gd_0.08_Bi_0.02_BO_3_ phosphor annealed at 800 °C exhibits the highest QY values of 24.75% and 1.33% under the excitation of 273 nm and 532 nm, respectively, confirming that the system possesses excellent excitability in both UV and VIS regions. The optimal QY from the Gd^3+^-Bi^3+^ co-doped YBO_3_ phosphor is 75 times the single Gd^3+^-doped sample. Bright and narrow UVB emissions resulting from efficient photon conversion demonstrate the multifunctional applications of Gd^3+^-Bi^3+^-activated YBO_3_ phosphors and provide a new route for skin treatments.

## Figures and Tables

**Table 1:** Quantum yields in Gd^3+^-Bi^3+^ co-doped phosphors with different Gd^3+^ contents and sintering temperatures under 273 and 532 nm excitation.

Excitation Wavelength (nm)	External Quantum Yield QY (%)									
	Gd^3+^ Content (x) 800 °C Annealing						Sintering Temperature (°C)			
	0	0.2	0.4	0.6	0.8	1.0	700	800	900	1000
**273**	0.33	7.53	14.80	21.81	24.75	3.91	13.70	24.75	14.02	12.83
**532**	0.01	0.51	0.91	1.25	1.33	0.23	0.81	1.33	0.77	0.76

## Data Availability

Data will be made available upon request.
